# Interphase chromosome positioning in *in vitro* porcine cells and *ex vivo* porcine tissues

**DOI:** 10.1186/1471-2121-13-30

**Published:** 2012-11-15

**Authors:** Helen A Foster, Darren K Griffin, Joanna M Bridger

**Affiliations:** 1 Laboratory of Genomic and Nuclear Health, Centre for Cell and Chromosome Biology, Division of Biosciences, School of Health Sciences and Social Care, Brunel University, Uxbridge,, West London UB8 3PH; 2 School of Biosciences, University of Kent, Kent, CT2 7NJ, Canterbury

## Abstract

**Background:**

In interphase nuclei of a wide range of species chromosomes are organised into their own specific locations termed territories. These chromosome territories are non-randomly positioned in nuclei which is believed to be related to a spatial aspect of regulatory control over gene expression. In this study we have adopted the pig as a model in which to study interphase chromosome positioning and follows on from other studies from our group of using pig cells and tissues to study interphase genome re-positioning during differentiation. The pig is an important model organism both economically and as a closely related species to study human disease models. This is why great efforts have been made to accomplish the full genome sequence in the last decade.

**Results:**

This study has positioned most of the porcine chromosomes in *in vitro* cultured adult and embryonic fibroblasts, early passage stromal derived mesenchymal stem cells and lymphocytes. The study is further expanded to position four chromosomes in ex vivo tissue derived from pig kidney, lung and brain.

**Conclusions:**

It was concluded that porcine chromosomes are also non-randomly positioned within interphase nuclei with few major differences in chromosome position in interphase nuclei between different cell and tissue types. There were also no differences between preferred nuclear location of chromosomes in *in vitro* cultured cells as compared to cells in tissue sections. Using a number of analyses to ascertain by what criteria porcine chromosomes were positioned in interphase nuclei; we found a correlation with DNA content.

## Background

Studying non-random positioning of chromosome territories in interphase nuclei has led to an understanding of the spatial control of gene expression, in addition to gene and regulatory element sequence and chromatin modification [1,2]. Meaning that the position of a chromosome territory within an interphase nucleus may contribute to the control of gene expression [3,4]. In our studies of human tissue culture cells we have demonstrated that chromosome positioning is correlated with mostly with gene density in young proliferating cells [5-7], which changes to a size associated distribution of chromosome territories once the cells have exited the cell cycle [7-10]. It could be further hypothesised that specific chromosomes and/or gene loci would change their nuclear location before or after changes in regulation associated with cell differentiation. Indeed there is evidence in the literature that interphase positioning of chromosome territories may be tissue-specific and alter after differentiation. In human tissue culture cells the interphase positioning of most human chromosomes is conserved in both skin fibroblasts and lymphoblasts with the exception of two chromosomes, 8 and 21 [6]. Cell types derived from similar differentiation pathways such as small lung cells and large lung cells, or lymphocytes and myleoblasts also exhibited similar overall chromosome positioning but with some distinct tissue-specific chromosome locations apparent between the different cell types [11]. Human and porcine chromosomes have also been demonstrated to change nuclear location after induced adipogenesis [12,13], and during normal spermatogenesis [14].

The majority of studies on chromosome positioning have focussed on human cells however it appears that in all species so far studied, that the positioning of chromosome territories is non-random [15], including human [5,6,16,17], birds [18,19], snail [20], mouse [21], cow [22], and non-human primates [23-26]. Even in lower organisms such as Hydra and bacteria – distinct organisation of the genome has been observed [27,28].

To date, there have not been many chromosome positioning studies on porcine cells despite the pivotal role of pigs. Indeed the pig has much to offer as a model for human disease, for example, in diabetes and obesity, fertility, infectious disease resistance and maternal aggression. The pig is an important organism given its physiological similarities to human and its agricultural significance [29]. Pigs and humans also share numerous similarities with respect to their genomes with comparable genome sizes [30], karyotypes [31], and synteny [32,33] and the first draft of the porcine genome sequence is now near completion. Studies so far have positioned chromosomes in porcine spermatozoa [14], mesenchymal stem cells and differentiated adipocytes [13] and resting and activated neutrophils [34]. Many of these studies however are on single or small numbers of cell types and have yet to provide much insight into what properties or characteristics are involved in porcine genome behaviour in interphase nuclei. Nonetheless, they have all revealed that chromosome behaviour in pig cells is responsive to external stimuli during proliferation or differentiation.

In this study, we have assessed the interphase genome behaviour of individual porcine chromosomes in a range of porcine cells and tissues. Such studies will allow us to understand more about the spatial influences on genome function in differentiation, development and disease. Thus, in order to understand more about porcine genome behaviour, we have positioned individual whole chromosomes in different cell types such as a newly generated adult porcine fibroblast line (SOB), an embryonic fibroblast cell line (ESK4), *ex vivo* lymphocytes and *ex vivo* bone marrow Mesenchymal stem cells (MSCs). Further, we have then developed methods to permit the 3D positioning of chromosome territories in frozen porcine tissue sections. This has allowed a comparison between tissues derived from different germ layers in the embryo; and a further comparison between cells grown in tissue culture and cells directly from the animal.

## Results

### Interphase chromosome positioning in porcine tissue culture cells

**Figure 1:**
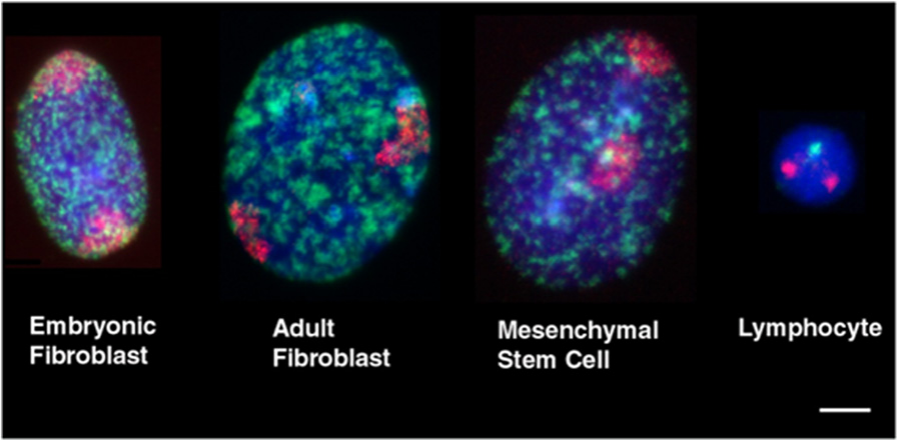
**Representative images of porcine chromosome territories in nuclei derived from embryonic and adult fibroblasts, mesenchymal stem cells (MSCs) and lymphocytes.** Chromosome territories are delineated with biotinylated whole chromosome painting probes amplified by degenerate oligonucleotide primed PCR (DOP-PCR) and visualised via strepavidin conjugated to cyanine 3 (red), DNA synthesis in S-phase nuclei is revealed by BrdU incorporation and indirect immunofluorescent detection with an antibody conjugated to FITC (green) and the DNA within the interphase nuclei stained with the DNA intercalator dye DAPI (blue). The lymphocyte image depicts dual-coloured FISH with the single Y chromosome labelled with digoxigenin and visualised by an anti-dig antibody conjugated to FITC. Bar, 10 μm.

**Figure 2:**
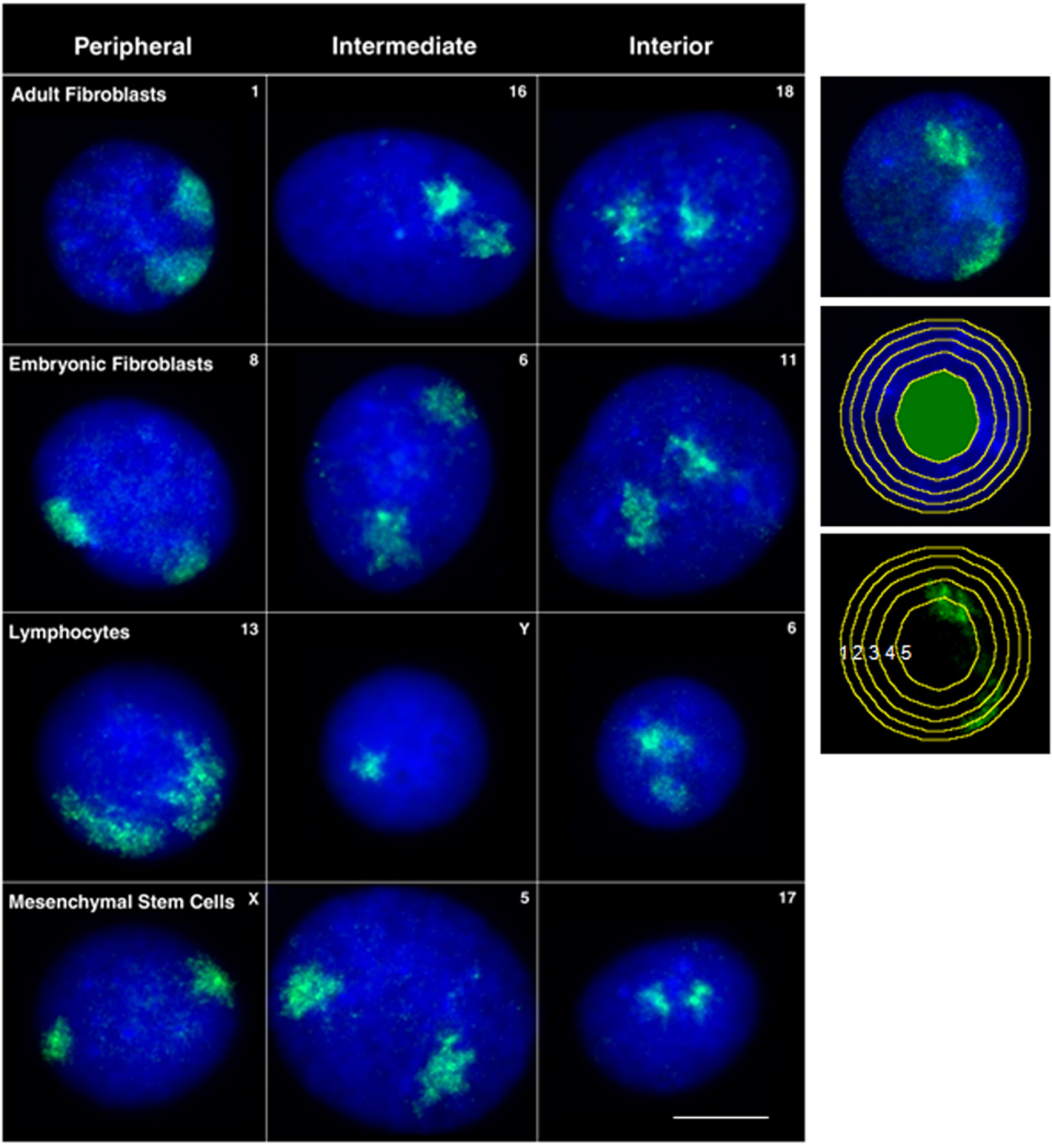
**Representative 2D FISH images displaying examples of peripheral, intermediately and internally positioned chromosome territories within (SOB), (ESK4), MSCs and lymphocytes.** Whole chromosome painting probes were labelled with biotin and detected using streptavidin conjugated to Cy3 (pseudocoloured green) and the nuclei were counterstained with DAPI (blue). The number/letter on the top right hand-side of each image depicts the chromosome painted in each nucleus. Bar, 10 μm.The right hand panel demonstrates how the erosion analysis works by delineating the DAPI mask and then eroding in revealing five shells of equal area in which the % signal of DAPI and the chromosome signal are measured.

**Figure 3:**
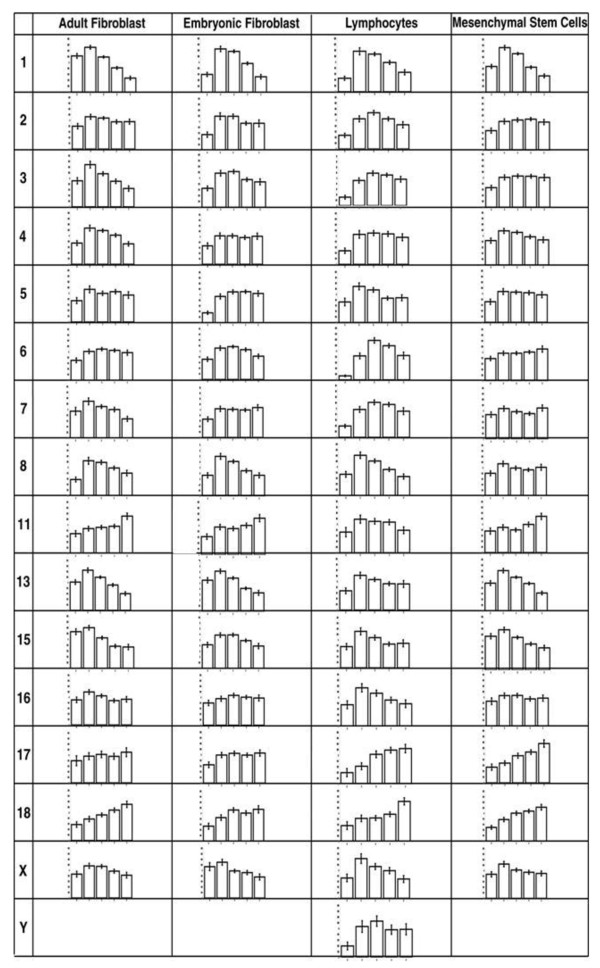
**Radial positioning of chromosome territories determined via two-dimensional FISH in adult fibroblasts (SOB), embryonic fibroblasts (ESK4), and MSCs during S-phase and in lymphocytes.** Erosion analyses were performed by ascertaining the distribution of the mean proportion of hybridisation signal per chromosome (%), normalised by the percentage of DAPI signal, over five concentric shells of equal area from the nuclear periphery to centre [5]. The x-axis displays the shells from 1–5 (left to right), with 1 being the most peripheral shell and 5 being the most internal shell. The y-axis shows signal (%)/ DAPI (%), from 0 to 1.8 with 0.2 increments.

Interphase chromosome positioning for nearly all porcine chromosomes was assessed in two lines of porcine fibroblasts derived from adult and embryonic tissue, mesenchymal stem cells derived from porcine bone marrow, and lymphocytes directly isolated from porcine blood (Figure 1). Chromosome positions in human interphase nuclei are different in non-proliferating cells and can even be found in alternative locations reversibly quiescent or irreversibly senescent cells [8,9,35]. In order to be sure that the cells we were analysing were in the proliferative cell cycle, we used cells that were in S-phase due to a short pulse of BrdU for incorporation into newly synthesised DNA during DNA replication (Figure 1). This was not possible for lymphocytes as they were isolated directly from pig blood. Figure 2 displays representative images of chromosome territory delineation and position in the four analysed cell-types in 2D flattened proliferating nuclei. Fifty images of each cell type, with each chromosome paint, apart from SSC9, SSC10, SSC12, SSC14, and SSCY (in female primary cells) due to probe availability, were subjected to an erosion analysis script that divides the nucleus into five concentric shells of equal area (see [5]). The percentage of DNA signal intensity and the percentage of chromosome signal intensity were measured for each shell. To normalise the data the chromosome signal intensity measurement per shell was divided by the DNA intensity measurement of the same shell. The mean chromosome position was determined from the distribution of normalised hybridisation signal across the five concentric shells, with shell 1 being the nuclear periphery and shell 5 the nuclear interior. These data were plotted in histograms and their relative position in nuclei determined (Figure 3), such that where the histograms display more normalised signal in shells 1 and 2 we state that the chromosomes are located more towards the nuclear periphery. If the histograms display more normalised signal in shells 4 and 5 then we state that the chromosomes are more towards the nuclear interior. An intermediate positioning is revealed when shell 3 displays more normalised signal then other shells, giving a bellshaped curve. The distribution of chromosome signal using this method was fairly easy to categorise for a number of graphs but for others it was not so straightforward and thus we have a bimodal distribution for chromosome 5 in adult fibroblasts and equally distributed for chromosome 2 also in adult fibroblasts. The data for each specific shell from the different cell types was collated and compared using a one-way ANOVA (Table 1). The statistical analysis clearly shows no significant difference in the interphase positions of porcine chromosomes 2, 16, 18 and X in each of the cell types. Interestingly, when the lymphocyte data was removed from the one-way ANOVA test, there were also no significant differences for chromosomes 4, 6, 7, 11, and 13 (Table 2). It is notable that lymphocytes tended to have fewer chromosomes signal at the nuclear periphery (shell 1) compared with any other cell type. The statistical analyses shown in Tables 2 and 3 demonstrate that, in general, the spatial positioning of whole chromosomes between the different cell types is generally conserved. 2D chromosome territory positioning analyses were confirmed in 3D fixed culture cells with a sub-set of chromosome paints (data not shown).

**Table 1:** Statistical analysis of all chromosome positions by erosion shell

**Chr.**	**Shell**	**All culture cells**	**Chr.**	**Shell**	**All culture cells**
	1	S *P* = 8.3E-13		1	NS
	2	NS		2	NS
1	3	NS	11	3	NS
	4	NS		4	NS
	5	NS		5	S *P* = 0.015
	1	NS		1	S *P* = 0.009
	2	NS		2	NS
2	3	NS	13	3	NS
	4	NS		4	NS
	5	NS		5	NS
	1	S *P* = 3.96E-05		1	S *P* = 0.009
	2	S *P* =0.004		2	NS
3	3	NS	15	3	NS
	4	NS		4	NS
	5	S *P* =0.037		5	NS
	1	S *P* = 0.037		1	NS
	2	NS		2	NS
4	3	NS	16	3	NS
	4	NS		4	NS
	5	NS		5	NS
	1	S *P* = 0.013		1	NS
	2	NS		2	S *P* = 0.006
5	3	NS	17	3	NS
	4	NS		4	NS
	5	NS		5	NS
	1	S *P* = 5.57E-09		1	NS
	2	NS		2	NS
6	3	S *P* = 0.0007	18	3	NS
	4	NS		4	NS
	5	NS		5	NS
	1	S *P* =0.0002		1	NS
	2	NS		2	NS
7	3	NS	X	3	NS
	4	NS		4	NS
	5	S *P* = 0.03		5	NS
	1	NS			
	2	NS			
8	3	S *P* = 0.025			
	4	NS			
	5	S *P* = 0.023			

Statistical analysis of 2D FISH data using one-way ANOVA. Data for each specific shell from the different cell types (SOB, ESK4, lymphocytes and MSCs) was pooled and compared for each chromosome. Key: S –significant; NS – not significant.

**Table 2:** Statistical analysis of the interphase position of chromosomes 3, 4, 5, 6, 7, 8, 11 and 13 between cells types

**Comparison of data from statistically significant shells**								
	**Chromosome 3 Shell 2**			**Chromosome 3 Shell 5**			**Chromosome 7 Shell 5**	
	**Average**	**Variance**		**Average**	**Variance**		**Average**	**Variance**
SOB	**1.503455**	**0.864878**	SOB	**0.624348**	**0.499283**	SOB	**0.666543**	**0.43894**
ESK4	1155806	0.581671	ESK4	0.879521	0.713582	ESK4	1.068023	0.691955
Lymp	0.921049	0.511082	Lymp	0.966148	0.606659	Lymp	0.924432	0.833826
MSC	1.022304	0.497753	MSC	1.018609	0.761672	MSC	1.077205	0.744537
	*P* = 0.2 (ESK4, Lymph,MSC) data)			*P* = 0.6 (ESK4, Lymph, MSC) data)			*P* = 0.56 (ESK4, Lymph, MSC) data)	
	**Chromosome 5 Shell 1**			**Chromosome 4 Shell 1**			**Chromosome 6 Shell 1**	
	**Average**	**Variance**		**Average**	**Variance**		**Average**	**Variance**
SOB	0.754016	0.693435	SOB	0.671767	0.743471	SOB	0.711614	0485381
ESK4	**0.356924**	**0.215757**	ESK4	0.756511	0.500148	ESK4	0.723531	0.381516
Lymp	0.721551	1.078762	Lymp	**0.469425**	**0.507708**	Lymp	**0.143052**	**0.036936**
MSC	0.721894	0.611738	MSC	0.851697	0.542092	MSC	0.795698	0.377657
	*P* = 0.97 (SOB, Lymph, MSC) data)			*P* = 0.4 (SOB, Lymph, MSC) data)			*P* = 0.7 (SOB, Lymph, MSC) data)	
	**Chromosome 7 Shell 1**			**Chromosome 11 Shell 5**			**Chromosome 13 Shell 1**	
	**Average**	**Variance**		**Average**	**Variance**		**Average**	**Variance**
SOB	0.947942	0.931489	SOB	1.302983	0.873085	SOB	1.004326	0.452558
ESK4	0.672041	0.457591	ESK4	1.249509	1.015762	ESK4	1.119332	0.505126
Lymp	**0.391862**	**0.193191**	Lymp	**0.802752**	**0.833718**	Lymp	**0.684924**	**0.604833**
MSC	0.879727	0.596214	MSC	1.295503	0.841152	MSC	0.967014	0.489344
	*P* = 0.14 (SOB, ESK4, MSC) data)			*P* = 0.9 (SOB, ESK4, MSC) data)			*P* = 0.44 (SOB, ESK4, MSC) data)	
	**Chromosome 8 Shell 3**			**Chromosome 8 Shell 5**				
	**Average**	**Variance**		**Average**	**Variance**		**Average**	**Variance**
SOB	1.196292	0.300132	SOB	0.802398	0.568513			
ESK4	1.227353	0.227939	ESK4	0.733376	0.505745			
Lymp	1.216679	0.296789	Lymp	1.019978	0.775982			
MSC	**0.984072**	**0.316611**	MSC	**0.66373**	**0.485217**			
	*P* = 0.94 (SOB, ESK4, Lymp) )data)			*P* = 0.6 (SOB, ESK4, Lymp) data)				

Table shows detailed data from the statistically significant nuclear shells. The cell type that causes the statistical difference is highlighted accordingly in bold. The *P* value shows comparisons with each cell types excluding the statistically different shell using one-way ANOVA.

**Table 3:** Predicted positions of chromosome position according to various parameters compared to actual distribution

**Chromosome**	**Actual porcine chromosome territory position**	**Analogous human chromosome territory position**^**1**^	**Human epigenetic markers**^**2**^	**Porcine epigenetic markers**^**3**^	**Predicted*in silico*porcine gene density**^**4**^	**Size**^**5**^
1	P	P	I	P	I	P
2	I	I	I/Int	Int	Int	P
3	I	I	I	I	I	I
4	I	I	I	P	I	I
5	I	P	Int	P	I	I
6	I	Int	Int	Int	I	P
7	I	Eq	Int	Int	I	I
8	P	P	P	P	P	I
11	Int	P	P	P	P	Int
13	P	P	P	Int	P	P
15	P	P	P	P	I	P
16	Eq	I	P	P	I	Int
17	Int	Eq	Int	Int	I	Int
18	Int	P	Int	P	I	Int
X	P	P			I	I

This table displays comparisons between the chromosome territory positioning of actual porcine chromosomes from 2D FISH in SOB cells relative to predictions from synteny with human chromosomes, epigenetic markers, *in silico* gene data and chromosome size. This allows a comparison to show how the positioning of whole porcine chromosomes within the nucleus fit with the size and gene density theories of organisation. 1 Synteny between the human and porcine genome (https://wwwlgc.toulouse.inra.fr/pig/compare/SSC.htm, see also http://piggenome.org/docs/index.php) was used to predict the analogous porcine chromosome territory position from human chromosome territory data [6]. 2 The assignment of human epigenetic markers (e.g. CpG island-rich chromatin, early replicating DNA, late replicating DNA) [42,53] can be used through synteny studies as markers to predict the gene density of chromosomes. A projection of chromosome territory positioning can be made according to the gene-density theory of organisation, since more gene-rich chromosomes would be expected to occupy internal nuclear positions, and gene-poor chromosomes having more peripheral localities. 3 Porcine epigenetic markers (e.g. CpG island-rich chromatin and H3-isochore rich chromatin) were used to predict potential chromosome positioning according to the gene density theory of organisation. The last two columns show the chromosomes and their categorised nuclear locations in order of gene density and size. Key, P – peripheral; I – intermediate; Int – interior; Eq – equally distributed.

### Chromosome positioning in 3D *ex vivo* tissue sections

The cultured cell types used to investigate chromosome territory organisation were all derived from mesodermal origin, and thus followed a similar differentiation pathway. This may account for the similarities in analogous chromosome territory positioning between the different cell types analysed. To investigate how genome organisation is influenced in cell types derived from divergent differentiation pathways, chromosome positioning was ascertained in tissues, namely kidney (mesodermal), lung (endoderm), and brain (ectodermal) (Figure 3).

**Figure 4:**
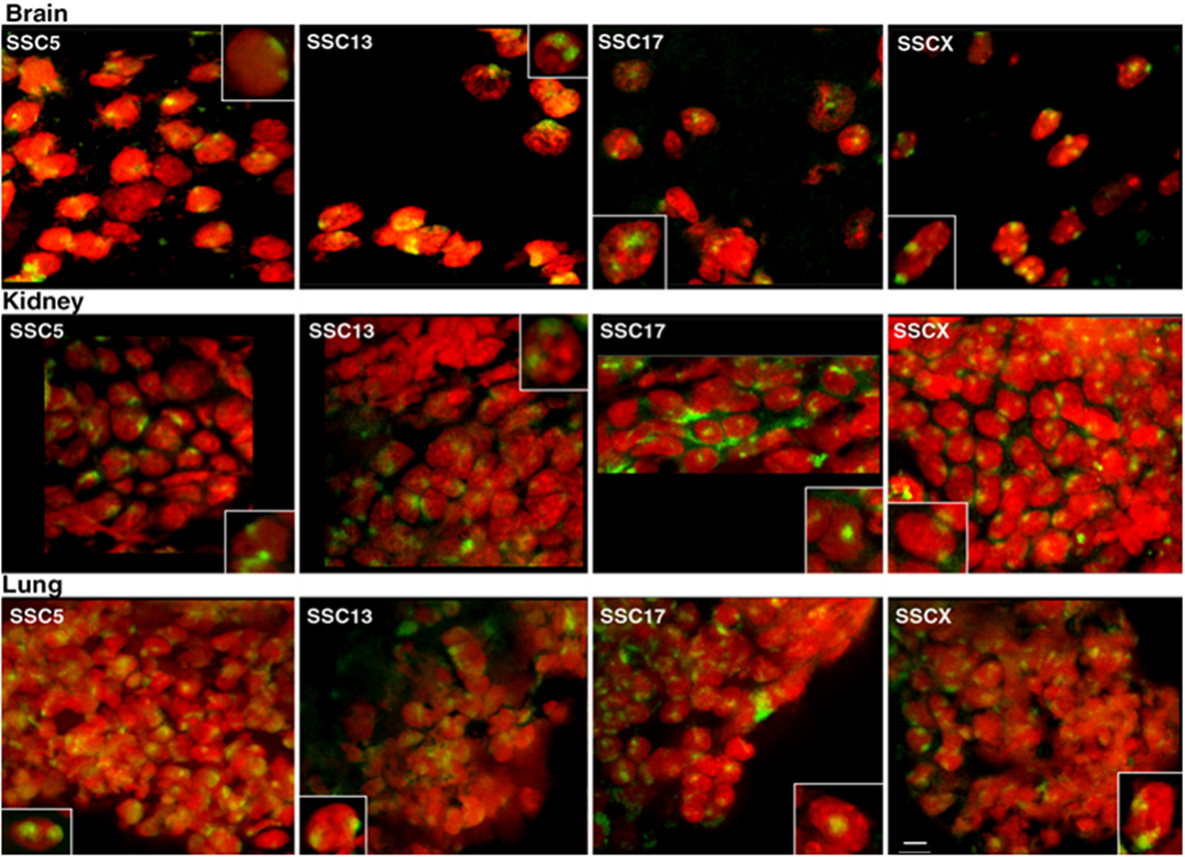
**Confocal optical sections displaying the nuclear positioning of chromosomes 5, 13, 17 and X in porcine brain, kidney, and lung tissue.** The insets show nuclei that have been enlarged for greater detail from other fields of view. Whole chromosome painting probes were labelled with biotin and detected using streptavidin conjugated to fluorescein isothiocyanate (green) and the nuclei were counterstained with propidium iodide (red). Bar, 10 μm.

**Figure 5:**
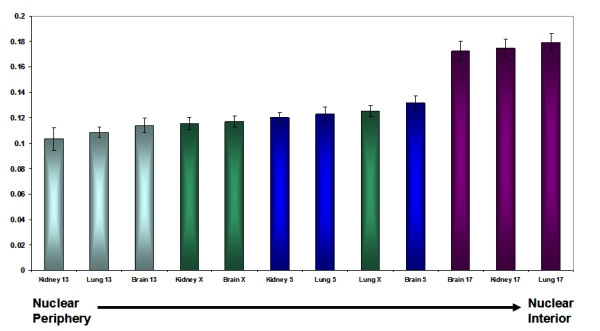
**Histogram showing the radial positioning data for chromosomes 5, 13, 17 and X territories in tissue sections of brain, kidney, and lung using whole chromosome painting probes.** The centre of each chromosome territory to the nearest nuclear edge (in 3 dimensions) was measured and normalised by the length measurement for each nucleus.

The nuclear positioning of chromosomes 5, 13, 17 and X was analysed. These chromosomes were chosen to represent different sizes of chromosome i.e. SSC 13 (large) SSC 17 (small) and SSC 5 and X for their behaviour in other cells i.e. SSC5 was found in a different nuclear position in each cell-type whereas X was always at the nuclear periphery. New methods were developed to perform 3D-FISH with whole chromosome painting probes on porcine tissue. Optical sections of the tissue were captured using confocal microscopy (Figure 4) and the digital images analysed in Imaris. The distances of the centre of the chromosome territory to the nearest nuclear edge were measured and this measurement was normalised by the longest width of the nucleus. Figure 5 demonstrates the relative nuclear position of these four chromosomes. The small chromosome 17 had a more intermediate/internal position in all of the tissue types examined. The other chromosomes, which include the large chromosomes 13 and the intermediate sized chromosomes X and 5, were all located towards the nuclear periphery. This was confirmed via statistical analysis using an unpaired, unequal variance, two-tailed Student t-test. Comparison of the chromosome positions between the different tissue types, at the 95% confidence level showed no statistical difference in the nuclear positioning of chromosome territories for 5, 13, 17 or X in brain, kidney, or lung tissue sections.

## Discussion

Attention has been focused towards mapping the position of whole porcine chromosome territories in interphase nuclei in a quest to understand further how and why the genome is organised in the way that it is, within the nuclear environment. In this study, the position of porcine chromosomes within interphase nuclei from a number of cell types was mapped and correlated to both gene density and size theory of chromosome positioning.

This study has determined that, like many other genomes studied, the porcine genome is highly organised into territories that are non-randomly positioned within the nucleus. Indeed, this is the largest study so far to investigate porcine genome organisation and the nuclear positioning of porcine whole chromosomes. This study has mapped the position of the majority of porcine whole chromosome territories in a number of different cell types, surpassing that of any other species. It remains unclear however how chromosomes assume these nuclear positions.

**Figure 6:**
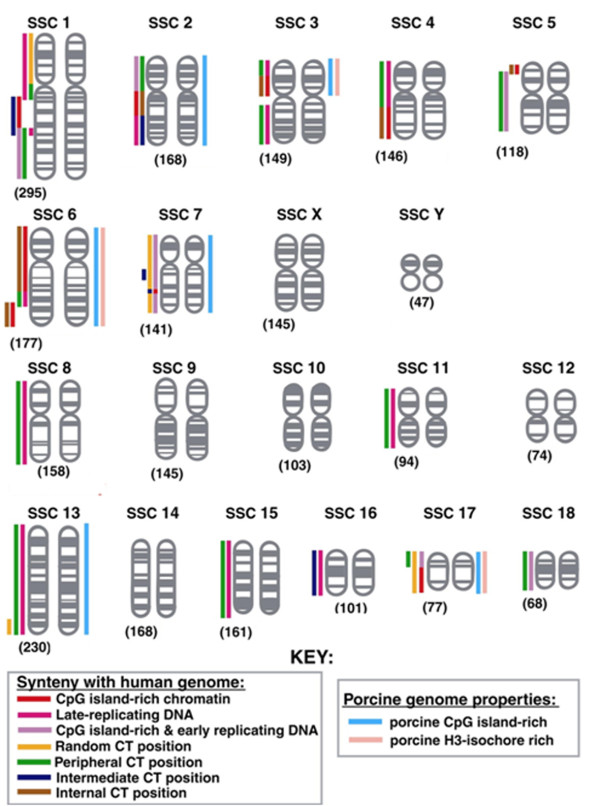
**Schematic diagram showing the porcine karyotype.** Each chromosome has its size represented (Mbp) [52]. Porcine CpG island [39] and H3-rich isochore distribution [40] is shown on the right-hand side of each chromosome. Synteny to human CpG island distribution and early replicating DNA [42], synteny to late replicating human DNA [42], and synteny to human nuclear chromosome positions [6] are shown of the left-hand side of each chromosome. This was performed by examining syntenic regions between the porcine genome and human genome (https://wwwlgc.toulouse.inra.fr/pig/compare/SSC.htm - last accessed 26.08.10; see also http://piggenome.org/docs/index.php). Synteny for chromosomes 9, 10, 12, 14 were not performed since we did not have flow-sorted template for these chromosomes to make chromosome painting probes. Therefore we have no data for these chromosomes.

Existing evidence shows that there are correlations between the nuclear positioning of a chromosome and its gene density [5,6,36] or size [8,17,37,38]. We have, for the purposes of this study, attempted to calculate the gene density of each chromosome via the distribution of porcine CpG islands [39], H3 isochores [40], using synteny to the human genome with respect to CpG island distribution, early and late replicating DNA and the known nuclear positioning of syntenic chromosomes (Figure 6 and Table 3) [5,6,41-43] and via the current gene assignments mapped to porcine chromosomes on e!Ensembl (http://www.ensembl.org/Sus_scrofa/Location/Genome - last accessed 26.08.10) (Table 4), it should be noted that the genome has not yet been fully annotated. Table 3 displays our predicted porcine chromosome territory interphase positioning according to these various estimates of gene density of each porcine chromosome or known positioning of human chromosomes in proliferating fibroblasts or lymphoblasts. With these correlations, less than half of the porcine chromosomes actual positions fit with the predicted positions. Although the porcine genome has been sequenced, the assignment of a number of genes still needs to be determined.

**Table 4:** **A comparison between the chromosome territory positioning of actual porcine chromosomes from 2D FISH studies with respect to synteny with human chromosomes, porcine epigenetic markers,*in silico*gene data and chromosome size**

	**1**	**2**	**3**	**4**	**5**	**6**	**7**	**8**	**11**	**13**	**15**	**16**	**17**	**18**	**X**
**Chromosome size (Mbp):**	295	168	149	146	118	177	141	158	94	230	161	101	77	68	145
**Sequenced Length (Mbp):**	295	140	124	136	101	123	136	120	80	145	135	77	64	54	126
**Known Protein-coding Genes:**	80	56	32	40	39	46	125	20	3	29	13	8	28	9	17
**Novel Protein-coding Genes:**	1,716	1,642	1,070	1,012	923	1,304	1,463	552	289	843	698	326	532	355	684
**Pseudogene Genes:**	62	31	23	28	22	22	54	20	6	32	23	4	16	11	32
**miRNA Genes:**	68	45	27	26	27	46	50	21	17	30	19	19	17	23	75
**rRNA Genes:**	12	8	10	16	10	11	11	5	7	10	6	7	6	3	9
**snRNA Genes:**	106	44	52	51	47	69	60	38	29	60	55	42	28	27	60
**snoRNA Genes:**	65	34	30	26	20	41	52	20	10	31	28	9	10	5	26
**Misc RNA Genes:**	20	11	7	14	5	12	10	10	6	9	6	2	2	3	8
**Total number of genes**	2129	1871	1251	1213	1093	1551	1825	686	367	1044	848	417	639	436	911
**Predicted gene density**	7.2	11.1	8.4	8.3	9.3	8.8	12.9	4.3	3.9	4.5	5.3	4.1	8.3	6.4	6.3

*In silico* information with the current (as of September 2010) number of genes mapped and predicted for the porcine genome derived from e!Ensembl (http://www.ensembl.org/Sus_scrofa/Location/Genome). Each gene category has been added together to show the total number of genes assigned to each chromosome. The table shows individual porcine chromosome size in Mbp [52] and length sequenced in Mbp. The predicted gene density of each chromosome was calculated by: the total number of genes/chromosome size. The higher the gene density value, the more gene dense the chromosome.

Using the latest porcine genome information we have calculated a gene-density value for each chromosome by dividing the possible genes on each chromosome by the Mbp size for that chromosome. These values range from 3.9 for porcine chromosome 11 to 12.9 for chromosome 7 (Table 4). In Table 3 we display the chromosomes and their categorised position in SOB adult fibroblasts in descending order of gene density and the correlation between gene density and position is weak with the poorest gene dense chromosome being in the nuclear interior and the richest at the nuclear periphery. Given that the genome sequencing is only near completion there may still be a gene density distribution that presently remains elusive. However, the correlation appears much more convincing for size with the three smallest chromosomes being found in the nuclear interior and the two largest chromosomes at the nuclear periphery. It should also be noted that these are proliferating cells but they are all in S-phase. For humans no difference in chromosome positioning for human chromosomes 18 and 19 was revealed in S-phase [6].

Our studies allowed comparison in the positioning of homologous chromosomes in kidney fibroblasts derived from both adult and embryonic tissue. It was revealed that chromosomes had comparable interphase nuclear locations. Since cells from the embryonic lineage had already differentiated to kidney fibroblasts and followed the same differentiation pathway as the adult kidney fibroblasts, then similar chromosome positioning was perhaps likely.

The more interesting comparison perhaps is the comparison between the *ex vivo* lymphocytes and the tissue culture cells, since lymphocytes are derived from haemopoietic lineage and therefore may also exert distinct tissue specific control of genome organisation. Despite over 95% of cultured lymphocytes being deficient in A-type lamins and the difference in stem cell lineage, the majority of chromosomes occupied similar nuclear locations. We did however note that we found less chromosome signal at the nuclear periphery in shell 1 for lymphocytes, which may be due to the role A-type lamins play in anchoring the genome to the nuclear periphery [44]. However, the amount of total chromatin as stained by DAPI was not dissimilar to other cell types used in this study.

The use of pig tissues and development of a 3D FISH protocol has allowed us also to perform pig chromosome territory delineation and position analysis in cells in 3D preserved frozen tissue sections. So to determine whether the spatial positioning of chromosomes within nuclei is tissue specific, the nuclear position of four porcine chromosomes 5, 13, 17 and X were ascertained in three different tissues types including brain, kidney, and lung. Although each tissue type arose from different origins (endoderm, mesoderm or ectoderm) and contained specialised cells, analogous porcine chromosomes occupied equivalent interphase nuclear positions in each of the tissue types analysed. These data differed to a previous study that investigated genome organisation in nuclei derived from specific mouse tissues [11]. Parada and colleagues found that although some chromosomes shared similar interphase nuclear positions, many exhibited a tissue specific organisation, particularly cells that followed divergent differentiation pathways [11].

From this study it is apparent that chromosomes occupy similar interphase nuclear positions regardless of *in vitro* or *in vivo* conditions. Comparisons between chromosome positioning of chromosomes 5, 13, 17 and X in cultured nuclei (during S-phase) and nuclei within *ex vivo* tissue sections, reveals that chromosomes 5, 13 and X occupy peripheral positions, whereas chromosome 17 is localised at more internal nuclear locations (Figure 5). Nuclear positioning data for chromosomes 5, 13, 17 and X verify that chromosomes share analogous nuclear positions between *in vitro* cultured nuclei and *in vivo* nuclei. However, variations in culture conditions, such as serum starvation, can substantially affect relative interphase chromosome positioning as demonstrated with human chromosomes becoming repositioned from a peripheral nuclear location to an internal location upon quiescence [9]. Considering the possible influence of altered culture conditions on genome organisation, serum was removed from adult porcine fibroblasts cultures for several days in an effort to quiesce cells. Unfortunately, we were unable to quiesce the cells via a decrease in serum levels to 0.5% or via contact inhibition since apoptosis was induced and many cells were lost, a phenomenon previously reported in cultured pig cells [45]. Therefore we could not determine if chromosome territories were repositioned within the nuclei in quiescent porcine cells. Nevertheless, despite the proliferation status of *in vivo* cells in tissue sections being undetermined, equivalent chromosome positions within nuclei were exhibited regardless of their proliferation status. The brain is composed of two main cell types neurons and glial cells, with glial cells representing 90% of brain cells. Brain cells are terminally differentiated and have ceased cell division [46], however, despite their quiescent nature no alterations in the nuclear position of specific porcine chromosomes were ascertained. The conditions used to make *in vitro* cells quiescent, such as serum starvation or contact inhibition do not typically mimic *in vivo* conditions of quiescent cells. Thus, *in vitro* quiescent cells may exhibit altered chromosome territory position due to specific signal transduction pathways not normally activated or silenced in *in vivo* cells. Although the pig seems an excellent animal model it is also possible that species-specific differences may arise resulting in conflicting data. This is evident when using the mouse as a model for genome organisation. The mouse genome is uniform in size and gene density and chromosomes are positioned at different nuclear locations within mouse nuclei compared to the nuclear position of syntenic human chromosomes in human nuclei [47].

In summary, the porcine genome is non-randomly organised with most chromosomes occupying similar nuclear positions despite developmental origin or lineage and regardless of being cultured *in vitro* or from *in vivo* tissue. From epigenetic data, projected gene assignments and chromosome size, it would appear presently that the porcine genome does not fit completely into either the size or gene-density model of chromosome positioning. We will assess gene-density correlated positioning again in the future when the porcine genome is well established. Thus it appears that other parameters also influence the positioning of whole chromosomes within the nucleus.

## Conclusions

This study has established the pig as a model organism for genome organisation. The majority of porcine chromosomes have been positioned in a number of different cells types surpassing that of any other organisms. It has allowed comparison between *in vivo* and *ex vivo* material with respect to chromosome positioning and between cell types. It is thus an important study for the interphase genome organisation field but also for the porcine genome community.

## Methods

### Culture cell preparation

Porcine adult kidney fibroblasts, and mesenchymal stem cells were isolated from pig kidney and bone marrow samples respectively. MSCs were cultured in Dulbecco’s modified Eagles medium (DMEM) with 20% (v/v) NCS, antibiotics (10 units/ml penicillin, 50 μg/ml streptomycin) and 2 mM L-glutamine for approximately 3 days of culture and then the serum level was reduced to 10% [48]. The adult porcine kidney fibroblasts (SOBs) were cultured in Minimum Essential Medium Eagle (EMEM) medium containing Earle’s balanced salt solution (EBSS) (Sigma-Aldrich) and supplemented with 2 mM Glutamine, 1% non-essential amino acids, antibiotics (10 units/ml penicillin, 50 μg/ml streptomycin) and 20% foetal bovine serum. Again, after 3 days the serum level was lowered to 10%. All of the primary porcine cells were grown at 37°C with a 5% carbon dioxide level. The porcine embryonic kidney fibroblast (ESK4) cell line was purchased from European Collection of Cell Cultures [49]. Cells were cultured in EMEM (EBSS) medium (Sigma-Aldrich) supplemented with 2 mM Glutamine, 1% nonessential amino acids, antibiotics (10 units/ml penicillin, 50 μg/ml streptomycin) and 10% foetal bovine serum. Porcine whole blood was collected in a lithium heparin vacuette® tube and 0.5 ml of blood was cultured in 9.5 ml of PB-MAX™ Karyotyping medium (Invitrogen) at 37°C with 5% carbon dioxide for 72 hours.

Cultured cells for 2D and 3D FISH (excluding lymphocytes) were pulse labelled for one hour prior to hypotonic treatment and fixation with BrdU (Sigma-Aldrich). This was to detect cells synthesising DNA (S-phase). 1 μl of 30 mg/ml BrdU and 1 μl of 0.3 mg/ml of 5-fluoro-2’-deoxyuridine (FUrd) (Sigma-Aldrich) were added to each ml of media present in the flask for the 1 hour pulse.

### Preparation of cells for 2D-FISH

Cultured cells (with the exception of the lymphocytes) were harvested using 0.25% trypsin solution, and then the trypsin was neutralised by the addition of an equal volume of complete medium. Cells were washed with versene and incubated with hypotonic buffer (0.075 M KCl) for 15 min at room temperature before being centrifuged at 300–400 g for 5 minutes. Cells were fixed with ice-cold 3:1 (v/v) methanol/ glacial acetic acid added drop wise to the cell pellet and then incubated for at least 1 hour at 4°C. The cells were centrifuged at 300–400 g for 5 min and the process of fixative addition was repeated a further four times with a 5–15 minute incubation before centrifugation. The cell preparation was dropped onto slides.

### Tissue section preparation

Porcine brain, kidney, and lung tissue samples were used in this study. Tissue samples were cut into small pieces, frozen in a hexane bath and stored at −80°C. 60 μm sections of frozen tissue were cut using a cryomicrotome (Bright 5030 microtome) and adhered to slides coated with 3% 3-aminopropyltriethoxysilane (APES).

The tissue sections were fixed with 4% paraformaldehyde at room temperature for 10 minutes. The sections were washed in PBS and permeabilised in 0.5% saponin (w/v) and 0.5% Triton X-100 (v/v) in PBS for 25 minutes. They were then rinsed in PBS and incubated in 0.1 N HCl for 10 minutes before being rinsed again in PBS. The tissue sections were digested with 200 μg ml–1 RNase A at 37°C for 1 hour. These were then washed and stored in PBS until denaturation.

### Probe preparation

Whole porcine chromosomes were isolated by flow sorting chromosomes prepared from peripheral blood lymphocytes. The chromosome templates underwent primary and secondary amplification by performing degenerate oligonucleotide primed polymerase chain reaction (DOP-PCR) and were subsequently labelled with biotin-16-dUTP (Boehringer Mannheim) [50,51]. However, due to non-suppressed sequences on chromosome 18 after FISH a repetitive region of chromosome 14 was microdissected and used as suppression DNA with the chromosome 18 probe. 300 ng biotin-16-dUTP labelled chromosome paint (450 ng for 3D FISH on *ex vivo* tissue samples), 50 μg sheared porcine genomic DNA and 3 μg herring sperm were ethanol precipitated at −80°C for at least 1 hour before dissolving in hybridisation mixture (50% formamide, 10% dextran sulphate, 2x SSC and 1% Tween 20) at 50°C for a minimum of 2 hours prior to performing FISH. The probes were denatured for 5 minutes at 75°C and left for 1 hour at 37°C.

### 2D Immuno-FISH

The slide preparations were aged for one hour at 70°C prior to dehydration via an ethanol series (70%, 90% and 100%). Nuclei derived from cultured cells were denatured in 70% formamide, 2x SSC pH7.0 at 70°C for 2 minutes prior to immediate immersion in ice-cold 70% ethanol for 5 minutes and then subsequently through another ethanol series before being air-dried. The appropriate probe was applied to each slide and was covered with a 22x22 mm^2^ coverslip, sealed with rubber cement and left in a humidified chamber at 37°C overnight. On removal of the coverslips, the slides were washed in 50% formamide, 2x SSC pH 7.0 at 45°C, three times for a duration of 5 minutes each. Slides subsequently washed with 0.1x SSC prewarmed at 60°C, but placed in a 45°C water bath, three times, for 5 minutes each, before being transferred to 4x SSC, 0.05% Tween 20 for 15 minutes at room temperature. 150 μl of 4x SSC, 0.05% Tween 20 and 3% BSA blocking solution was applied to the slide and incubated for 20 minutes at room temperature. The excess was removed and a mixture containing 120 μg/ml streptavidin Cy3 (Amersham Biosciences), 4x SSC, 3% BSA and 0.05% Tween 20 was applied to each slide. The slides were incubated at 37°C for 30 minutes in darkness. Three washes in 4x SSC, 0.05% Tween 20 were performed at 42°C for 5 minutes each, prior to a brief wash in fresh deionised water. Preparations were incubated with monoclonal anti-BrdU antibody (Becton and Dickinson) diluted 1:100 with 1% NCS PBS for 30 minutes at 37°C. The slides were washed three times with PBS before application of secondary antibody donkey anti-mouse FITC (Jackson Laboratory) diluted 1:60 with 1% NCS PBS for 30 minutes at 37°C. The slides were washed three times with PBS and briefly with deionised water before mounting with Vectashield anti-fade mountant (Vectorlabs) containing DAPI as a counterstain.

### 3D FISH on ex vivo tissue sections

For tissue sections, 12 μl probe was applied to each tissue section and sealed with an 18x18 mm coverslip and rubber cement. Both the probe and tissue sample were denatured at 85°C for 6 minutes and left to hybridise at 37°C in a humidified container for 2 days. The post-hybridisation washes were performed as described previously with the following exceptions: the probe detection was performed using a 1:100 dilution of streptavidin conjugated with fluorescein (Amersham Biosciences) and the sections were mounted using Vectashield anti-fade mountant with propidium iodide (PI) (Vectorlabs). Extra PI was added to the mountant to give a final concentration of 7.5 μg μl^–1^.

### Microscopy and image analysis

Nuclei that were subjected to 2D FISH were examined with a Leica epifluorescence microscope (Leitz DMRB) and observed using a 100x oil immersion objective (Leica). Images were acquired with a CCD camera (Sensys, Photometrics) and were pseudocoloured using Smart Capture VP v1.4 (Digital Scientific Ltd, Vysis) providing merged colour images.

Approximately, 50–60 positively BrdU stained nuclei were analysed using a script developed by Paul Perry in IPLab software [5]. The scripts allowed erosion shell analysis, dividing the nuclei into five concentric shells of equal area from the nuclear periphery to the centre, removing a certain amount of background and measuring the proportion of DAPI and FITC (probe) signal within each shell. Initially, the DAPI image was segmented and its area determined, before the removal of background by detecting the mean FITC pixel intensity within the nuclei and subtracting it from the FITC image. The proportion of DAPI and FITC probe was ascertained for each segment and normalised by dividing the area signal by the nuclear area in pixels.

Tissue section images were acquired with a BioRad MRC 600 confocal laser scanning microscope. Stacks of optical sections were collected at 1.5 μm intervals through the tissue. The stacks were viewed and analysed in 3D reconstructions using Imaris software. Measurements were taken from the centre of a territory to the nearest edge and were normalised by dividing this value by the longest nuclear length.

### Statistical analysis

Statistical analyses on 2D FISH data were performed using one-way ANOVA. In this instance, the null hypothesis states that there is no statistical difference between the mean nuclear chromosome positions between the populations examined and is accepted if the P value is higher than P = 0.05. If the P value is lower than P = 0.05, then the result was deemed significant and the null hypothesis was rejected.

Statistical analyses on 3D tissue sections were performed using unpaired, unequal variance, two-tailed Student t-test. In this instance, the null hypothesis states that there is no statistical difference between the mean nuclear chromosome positions between the two populations examined. A two-tailed P value of less that P = 0.05 was deemed a significant result and the null hypothesis was rejected. A two-tailed P value of higher than P = 0.05 indicated that the results were not significant and the null hypothesis was accepted.

## Authors’ contributions

HAF performed all the experiments and data analysis, bioinformatics, protocol design and trouble-shooting as well as drafting the first copy of the manuscript. DKG was involved in the initial discussions and development of the project, corrected and made suggestions for manuscript improvement. JMB designed and developed the project and finalised the manuscript. All authors have read and ratified the final version.
